# Prevalence and determinants of human papillomavirus genital infection in men

**DOI:** 10.1038/sj.bjc.6600194

**Published:** 2002-03-04

**Authors:** S Franceschi, X Castellsagué, L Dal Maso, J S Smith, M Plummer, C Ngelangel, S Chichareon, J Eluf-Neto, K V Shah, P J F Snijders, C J L M Meijer, F X Bosch, N Muñoz

**Affiliations:** International Agency for Research on Cancer, 150 Cours Albert Thomas, F-69372 Lyon cédex 08, France; Institut Català d'Oncologia (ICO), Av. gran Via s/n, km. 2.7, E-08907 L'Hospitalet del Llobregat, Barcelona, Spain; IRCCS Centro di Riferimento Oncologico di Aviano, Via Pedemontana occidentale 12, I-33081 Aviano (PN), Italy; Philippine General Hospital, University of the Philippines, Taft Avenue, Manila 2801, The Philippines; Faculty of Medicine, Prince of Songkla University, Hat-Yai, Songkla, Thailand; Faculty of Medicine, University of São Paulo, Av. Dr Arnaldo 455, São Paulo - SP CEP 01246, Brazil; School of Hygiene and Public Health, The Johns Hopkins University, 615 North Wolfe Street, Baltimore, Maryland 21205, USA; Free University Hospital, Postbus 7057, NL-1007 MB Amsterdam, The Netherlands

**Keywords:** human papillomavirus, male genital tract, sexual habits, cervical carcinoma

## Abstract

Four-hundred-forty-five husbands of women with invasive cervical carcinoma, 165 of women with *in situ* cervical cancer, and 717 of control women (age range 19–82 years) were interviewed and a sample of exfoliated cells from the penis obtained in seven case–control studies conducted by the International Agency for Research on Cancer. The characteristics of human papillomavirus-positive and human papillomavirus-negative husbands were compared using odds ratios and 95% confidence intervals. Thirteen per cent of the husbands of control women, 18% of the husbands of women with invasive cervical carcinoma, and 21% of the husbands of *in situ* cervical carcinoma women were positive for penile human papillomavirus DNA. Human papillomavirus 16 was detected in 45 husbands, human papillomavirus 18, 31 or 33 in 19, and human papillomavirus 6/11 in 6, but the majority of human papillomavirus infection (158) was with other or unspecified human papillomavirus types. The same human papillomavirus type was seldom identified in both husband and wife. The strongest variation in penile human papillomavirus infection was by country, with percentages among the husbands of control women ranging between 3% in Spain and 39% in Brazil. Having had over 50 lifetime sexual partners, compared with only one, was associated with an odds ratio of 2.3.

*British Journal of Cancer* (2002) **86**, 705–711. DOI: 10.1038/sj/bjc/6600194 www.bjcancer.com

© 2002 Cancer Research UK

## 

The importance of the ‘male factor’ (Skegg *et al*, 1982) in the aetiology of cervical carcinoma (CC) in women was suggested years before the identification of a sexually-transmitted virus, human papillomavirus (HPV), as the central cause of these tumours (Dürst *et al*, 1983; Muñoz *et al*, 1992). A close correlation has been reported between the frequency of cervical and penile carcinoma in populations (Bosch and Cardis, 1990) and in individual couples (Smith *et al*, 1980).

The development of increasingly accurate assays for HPV detection has brought a clearer understanding of the prevalence of, and risk factors for, cervical HPV infection in women. Human papillomavirus DNA has been identified in 99% of CC specimens (Walboomers *et al*, 1999). Among women with a normal Pap smear, between a few per cent and more than 50% of women with a normal Pap smear harbour HPV DNA in their cervix, depending upon age and country (Herrero *et al*, 2000a; Woodman *et al*, 2001). Conversely, progress in understanding the prevalence and natural history of genital HPV infection in men has been limited. Most surveys of penile HPV infection so far have included a few hundred men at most and have generally been restricted to young individuals (Hippeläinen *et al*, 1993a,b,c; Baldwin *et al*, 2001; Kjaer *et al*, 2001; Lazcano-Ponce *et al*, 2001).

The present study examines the prevalence and determinants of penile HPV infection, according to PCR-based assays, among 1143 husbands of women enrolled in seven case–control studies coordinated by the International Agency for Research on Cancer (IARC).

## MATERIALS AND METHODS

The combined data included in these analyses were collected in five case-control studies of invasive cervical cancer (ICC) and two case-control studies of cervical carcinoma *in situ* (CIS) all carried out by IARC. The fieldwork was conducted from 1985 through 1993 in Spain (two studies), Colombia (two studies), Brazil, Thailand, and the Philippines. The detailed methods of each of these studies have been described elsewhere (Muñoz *et al*, 1992; Bosch *et al*, 1993; Eluf-Neto *et al*, 1994; Chichareon *et al*, 1998; Ngelangel *et al*, 1998; Castelssague *et al*, 2002). In brief, case women had newly diagnosed, histologically confirmed ICC or CIS. Control women were sampled from the general population in the two studies of ICC in Spain and Colombia (population-based studies) and from the same hospitals as the case women for the other studies (hospital- or clinic-based studies). In all studies, control women were frequency-matched to case women by age. All protocols were cleared by the IARC and the local ethical and research committees.

The subjects of the present report are the husbands or current stable partners of case and control women enrolled in these studies. Current partners (herein referred to as husbands) were defined as men having had regular sexual intercourse with the index women for at least six months, irrespective of whether they were married.

Of the 3790 women (1896 cases and 1894 controls) enrolled in the original case-control studies, 2800 (1329 cases and 1471 controls) reported having a living husband at study entry. Nine hundred and eighty-four (74%) husbands of case women and 937 (64%) husbands of control women were interviewed. A total of 807 (82%) husbands of case women and 717 (77%) husbands of control women provided penile cytological specimens, of whom 610 (76%) and 533 (74%) yielded a PCR-based valid HPV result, respectively. Among 610 husbands of case women, 445 were husbands of women with ICC and 165 of women with CIS. The median ages of the husbands of control women and of ICC and CIS cases were, respectively: 45 (range 19–82), 50 (range 22–79), and 38 (range 22–76) years. The husbands for whom HPV testing results were available did not differ from those who did not wish to be included in the study or from those who had an inadequate penile sample, with respect to various indicators of sexual behaviour; the mean number of lifetime sexual partners was in the 11-to-20 category in both participants and non-participants.

All participating husbands and wives were interviewed separately using a face-to-face, structured questionnaire administered by specially trained interviewers of the same gender as the interviewee. The questionnaire elicited detailed information on demographic and socio-economic characteristics and a complete lifetime history of the subject's sexual experiences including genital hygiene habits. The lifetime numbers of regular and casual sexual partners and of partners who were prostitutes were evaluated separately and added together to obtain the total number of lifetime sexual partners.

### Biological samples and HPV DNA detection

After interview, two samples of exfoliated cells were taken from the penis: one from the distal urethra, using a very thin wet cotton-tipped swab, and another one from the external surface of the glans and coronal sulcus, using a standard wet cotton-tipped swab. A smear was prepared for cytology and the remaining cells were eluted in phosphate-buffered saline, pelleted and stored at −20°C. Cervical exfoliated cells were also collected from the women as described for each study (Muñoz *et al*, 1992; Bosch *et al*, 1993; Eluf-Neto *et al*, 1994; Chichareon *et al*, 1998; Ngelangel *et al*, 1998).

The detailed protocols used for HPV DNA detection by PCR in the cervical and penile specimens were described elsewhere (Muñoz *et al*, 1992; Eluf-Neto *et al*, 1994; Ngelangel *et al*, 1998). Briefly, amplification of a fragment of the beta-globin gene served as an internal control for sufficiency of each specimen for amplification. The L1 consensus primers MY09–MY11 (Manos *et al*, 1989) were used for the samples collected in the Colombian and Spanish studies. For samples collected in the remaining studies, GP5/6 or GP5+/6+ (studies initiated after 1995, women only) general primers were used (van den Brule *et al*, 1990; de Roda Husman *et al*, 1995). The GP5+/6+ primers were modified from GP5/6 and provided an increased detection level mainly of less common HPV types that differ from the common HPV 6/11, 16, 18, 31 and 33 types (de Roda Husman *et al*, 1995). In the study of men, GP5/6 PCR products were assessed for HPV-positivity using a cocktail of HPV-specific probes and were further genotyped by hybridization of the PCR products with type-specific probes for six HPV types (16, 18, 31, 33, and 6/11) (van den Brule *et al*, 1990). Samples which were HPV-positive, but did not hybridise with any of the type-specific probes, were classified as ‘other or unspecified’. As HPV testing in a proportion of the study women was performed with GP5+/6+ PCR and included a higher number of type-specific probes (33 instead of 6) (Jacobs *et al*, 1995) than the assays used for the husbands, a higher proportion of multiple HPV infections emerged. Thus, in the evaluation of the agreement in positivity to HPV 16 and HPV 18 types within couples, 22 wives with multiple HPV infections were classified as 16 or 18 if such types were identified in addition to others.

### Statistical analysis

The simple kappa coefficient and corresponding 95% confidence interval (CI) were used to evaluate the agreement in HPV findings between husbands and wives. When the observed agreement exceeds chance agreement, kappa and CI are positive.

Associations with the presence of penile HPV infection were evaluated using unconditional multiple logistic regression models with maximum likelihood estimation of parameter values to obtain odds ratios (OR) and corresponding 95% CIs (Breslow and Day, 1980). All logistic regression models were adjusted for the husband's age, country, and, when indicated, case/control status of the female partner (i.e., controls, ICC cases, and CIS cases), and total number of lifetime sexual partners. Tests for linear trend of the ORs were performed giving an increasing score for each level of the categorised variable and fitting them in the model as continuous variables.

Heterogeneity among countries in the association between penile HPV infection and number of sexual partners was tested by comparing the difference between the −2 log likelihood of the model estimating a common OR and that estimating a specific OR for each group to the chi-square distribution with degrees of freedom given by the number of groups minus one. The results for total number of lifetime sexual partners are presented as a graph, plotting, for each country, the ORs as a black square, whose size is inversely proportional to the variance of the estimate. Diamonds are used to plot the summary OR for all studies together. The centre of the diamond represents the OR and the extremes the 95% confidence intervals.

All *P* values were derived from two-sided statistical tests.

## RESULTS

Thirteen per cent of the husbands of control women were found to have penile HPV infection, the most commonly identified types being HPV 16 (15 men), followed by 18 and 6/11 (five each) (Table 1

**Table 1:**
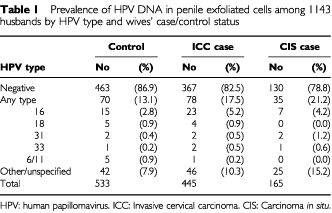
Prevalence of HPV DNA in penile exfoliated cells among 1143 husbands by HPV type and wives' case/control status

). Among the husbands of women with ICC and CIS, 17.5% and 21.2%, respectively, had penile HPV infection, most frequently HPV 16 (23 and seven husbands, respectively). Independently from the case/control status of the wife, the majority of penile infections involved ‘other or unspecified’ HPV types.

Figure 1

**Figure 1:**
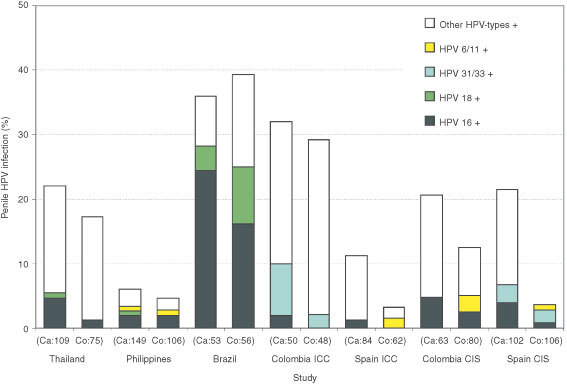
Prevalence of HPV DNA in penile exfoliated cells among 1143 husbands by wives' case/control status and study. ICC: Invasive cervical cancer. CIS: Carcinoma *in situ*. HPV: Human papillomavirus. Ca: Cases. Co: Controls.

shows the prevalence of penile HPV infection in each study according to wives' case/control status. The highest HPV prevalence was found in Brazil (36% and 39% among the husbands of case and control women, respectively), followed by the Colombia ICC study (32% and 29%, respectively). Studies from the Philippines (6% and 5% among the husbands of case and control women, respectively) and the Spain ICC study (12% and 3%, respectively) showed the lowest prevalence. The percentage of penile HPV infection attributable to HPV 16 ranged from less than 2% among husbands of cases and controls in the Philippines and in the Colombia and Spain ICC studies to 25% among the husbands of ICC cases in Brazil. Also, HPV 18 was more frequent in Brazil than elsewhere, while the highest percentage of HPV 6/11 was found among the husbands of controls in the study of CIS in Colombia (Figure 1).

The agreement in HPV-positivity within 964 couples is shown in Table 2

**Table 2:**
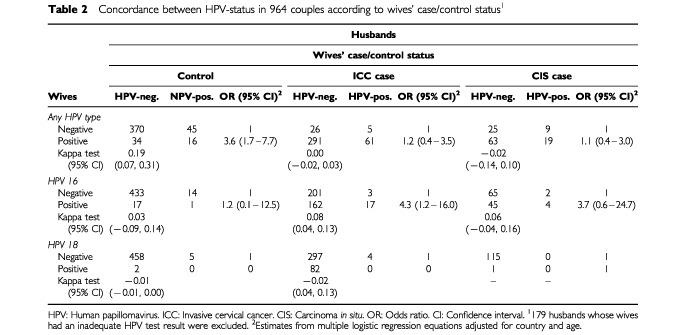
Concordance between HPV-status in 964 couples according to wives' case/control status^1^

for HPV of any type and for HPV types 16 and 18. The agreement in HPV infection of any type was better than chance only between control couples (kappa test=0.19; 95% CI: 0.07–0.31). The husbands of HPV-positive control women had a 3.6-fold (95% CI: 1.7–7.7) increased risk of being HPV-positive themselves, although not necessarily for the same HPV type. A four-fold increased risk of positivity for HPV 16 was found among the husbands of HPV-positive case women (Table 2). HPV 16 was concurrently found in 1/18 husbands of HPV-16 positive control women (kappa test=0.03; 95% CI: −0.09–0.14), in 17/179 husbands of ICC women (kappa test=0.08; 95% CI: 0.04–0.13), and in 4/49 husbands of CIS cases. HPV types 18 (Table 2), 31, 33, or 6/11 were found rarely in the husbands and never simultaneously in any couple. The agreement in HPV-positivity was not improved when the analyses in Table 2 were restricted to 124 couples where both husbands and wives reported only one sexual partner. HPV types 6/11, 16, 18, 31 and 33 were never found concurrently in monogamous husbands and wives (data not shown).

The risk of penile HPV infection was not associated with age or tobacco use after adjustment for country, wives' case/control status, and total number of lifetime sexual partners (Table 3

**Table 3:**
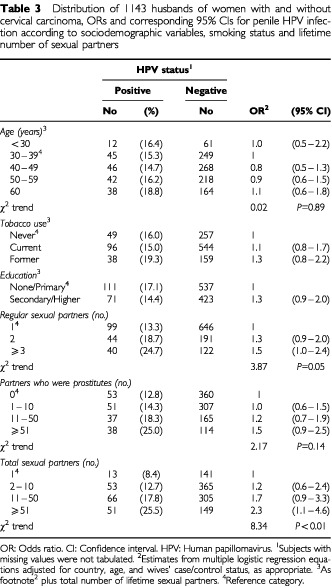
Distribution of 1143 husbands of women with and without cervical carcinoma, ORs and corresponding 95% CIs for penile HPV infection according to sociodemographic variables, smoking status and lifetime number of sexual partners

). Men who reported secondary education or higher showed an OR of 1.3 (95% CI: 0.9–2.0) compared with those who only attended primary school or were illiterate. The husbands' number of regular sexual partners (OR for ⩾3 *vs* 1=1.5), and of partners who were prostitutes (OR for ⩾51 *vs* 0=1.5) were directly correlated with the risk of penile HPV infection. For total number of lifetime sexual partners, the OR was 2.3 (95% CI: 1.1–4.6) for ⩾51 *vs* 1 partner (Table 3).

Age at first sexual intercourse, history of anal intercourse with women, sexual intercourse with men, and washing genitals regularly after sexual intercourse were unrelated to the risk of penile HPV infection (Table 4

**Table 4:**
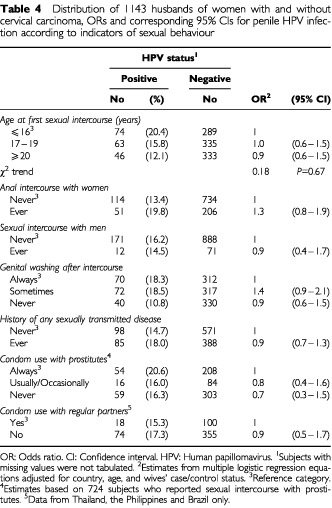
Distribution of 1143 husbands of women with and without cervical carcinoma, ORs and corresponding 95% CIs for penile HPV infection according to indicators of sexual behaviour

). Men who reported a positive history for any sexually transmitted disease (STD) had an OR for HPV infection of 0.9 (95% CI: 0.7–1.3) (Table 4). With respect to specific STDs, a history of syphilis was reported by 10 HPV-positive and 23 HPV-negative men (OR=1.8; 95% CI: 0.8–4.1). No association was found for history of gonorrhoea (OR=1.0; 95% CI: 0.6–1.5), genital herpes (OR=0.8; 95% CI: 0.3–1.8) or genital warts (OR=0.5; 95% CI: 0.2–1.3).

Regular use of a condom during intercourse with prostitutes was reported by 37% of husbands, with substantial variation between countries (1% in Colombia to 86% in Brazil). The OR among those who never used a condom with prostitutes *vs* those who always did was 0.7 (95% CI: 0.3–1.5) (Table 4), with no heterogeneity in the ORs between countries. The estimate was unchanged (OR=0.7; 95% CI: 0.2–3.3) when the analysis was restricted to the most promiscuous men (⩾11 sexual partners) (data not shown). Information on condom use with regular partners was available only for Thailand, the Philippines and Brazil. Seventy-eight per cent of husbands never used a condom with their regular partner (OR=0.9; 95% CI: 0.7–1.3).

Figure 2

**Figure 2:**
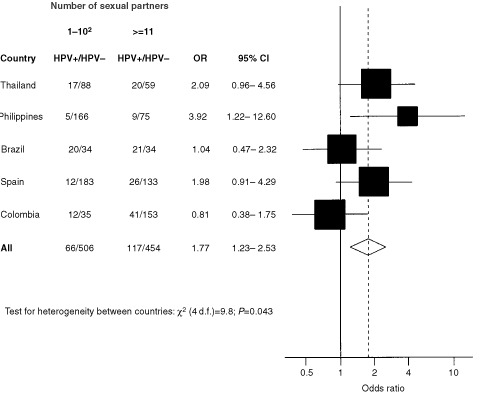
Distribution of 1143 husbands of women with and without cervical carcinoma, ORs and corresponding 95% CIs^1^ for penile HPV infection according to lifetime sexual partners and country. OR: Odds ratio. CI: Confidence interval. HPV: Human papillomavirus. ^1^Estimates from multiple logistic regression equations adjusted for age and wives' case/control status and country, where appropriate. ^2^Reference category.

shows the association between HPV penile infection and total number of sexual partners by country. The pooled OR for ⩾11 *vs* ⩽10 lifetime sexual partners was 1.8 (95% CI: 1.2–2.5), but there was heterogeneity between countries. In Colombia the OR (0.8; 95% CI: 0.4–1.8) was significantly lower than the pooled estimate and no increased OR was found in Brazil (OR=1.0). When the association with the number of sexual partners who were prostitutes was examined by country and overall (pooled OR for ⩾11 *vs* ⩽10 prostitutes=1.4; 95% CI: 1.0–2.0), again no risk increase emerged for Brazil (OR=1.0) or Colombia (0.9). Two regular partners or more, compared with one, was associated with a pooled OR of 1.4 (95% CI: 1.0–2.0), and in Brazil and Colombia with an OR of 1.1 (95% CI: 0.5–2.4) and 2.1 (95% CI: 1.1–4.0), respectively (data not shown).

## DISCUSSION

We have identified, by means of PCR-based assays, a prevalence of penile HPV infection among the husbands of women with or without cervical carcinoma ranging between 3 and 39%, depending upon country. Thus, the prevalence of HPV DNA in the male genital tract seems similar to that in the cervix uteri of control women in the same populations and age groups, if not higher (Muñoz *et al*, 1992; Eluf-Neto *et al*, 1994; Chichareon *et al*, 1998; Ngelangel *et al*, 1998). In agreement with findings in women without cervical neoplasia (Herrero *et al*, 2000b), HPV 16 and 18 types were relatively rare (i.e., less than one third of penile HPV infections) with the notable exception of Brazilian men, among whom these types accounted for the majority of penile HPV infections.

At least in our predominantly middle-aged population, the same type of HPV in both members was identified in only 2% of more than 1000 couples. With respect to HPV infections of any type, there was a moderately increased prevalence in the husbands of control women who were HPV-positive, but it is noteworthy that the most promiscuous men tended to be married to the most promiscuous women (Spearman correlation coefficient between lifetime sexual partners of wives and husbands=0.16). Ninety-two per cent of ICC female cases were positive for HPV DNA, yet penile HPV DNA was found in only 18% of the corresponding husbands.

A relatively poor correlation in HPV-positivity has also been reported in previous studies of women and their male sexual partners (Hippelaïnen *et al*, 1993c; Kyo *et al*, 1994; Baken *et al*, 1995; Strand *et al*, 1995; Castellsagué *et al*, 1997). The low agreement in our study may be partly due to technical reasons, since a relatively small amount of penile exfoliated cells could be obtained and fewer HPV types could be specifically identified in husbands than wives. Unlike cervical specimens, most penile ones could not be retested after new PCR-assays became available, on account of the low cell yield. However, a subset of male samples from the Philippines that were originally negative by GP5/6 PCR and could be retested, did not reveal additional positive findings by GP5+/6+ PCR (data not shown).

In some couples, the partner who has been sampled might not have been the relevant one. Agreement in HPV findings, however, was also modest in 124 couples where both the wife and husband reported only one lifetime sexual partner. The timing of the sampling of penile and cervical specimens at a relatively old age is, however, the likeliest explanation for our findings since spontaneous regression of HPV infection is common in men (Hippelaïnen *et al*, 1993b; Kjaer *et al*, 2001) and in women (e.g., control women in our study) who do not develop cervical neoplasias (Woodman *et al*, 2001). Among women with ICC or CIS, the relevant infection must have occurred many years earlier, and the relatively low prevalence of penile HPV infection in their husbands suggests that viral shedding of advanced cervical lesions is limited.

As in a few previous reports (Hippelaïnen *et al*, 1993c; Baldwin *et al*, 2001), the husbands in our study did not show, overall or in specific countries, clear variations in the prevalence of HPV penile infection by age. We were not able, however, to exclude the presence of an early peak of HPV infection (30–40%) in very young men at sexual debut (e.g., among conscripts in Denmark and Mexico) (Kjaer *et al*, 2001; Lazcano-Ponce *et al*, 2001). If the similarity of HPV penile infection across a broad age range is confirmed in future studies, this may suggest that spontaneously regressing infections in men are substituted by new ones over time.

When we looked at the correlates of penile HPV infection, most of the examined factors seemed to have no influence, with the exception of an elevated number of sexual partners. Prostitutes did not seem to be associated with a higher risk of penile HPV infection than other women. Furthermore, the association of HPV penile infection and number of sexual partners was not found in the two countries with the highest HPV prevalence in men, namely, Brazil and Colombia. In Colombia, although not in Brazil, the risk of penile HPV infection was found to be directly associated with the number of regular partners. The greater difficulty in elucidating the role of sexual habits in cervical cancer in high-risk (Muñoz *et al*, 1996) than in low-risk areas (Bosch *et al*, 1996) has already been discussed. Where, for reasons which are still unclear, the background prevalence of HPV genital infection in a population is very high, the probability of being infected seems already elevated at a relatively low level of sexual promiscuity.

In our study, condoms were seldom used as a contraceptive method with regular sexual partners, but relatively often with prostitutes. Use was not associated with a decreased risk of penile HPV infection. Although a condom confers substantial protection against several STDs (e.g., gonorrhoea, human immunodeficiency virus, etc.) (Cates and Stone, 1992), a favourable effect against HPV infection in women or men has not been shown so far (Syrjanen *et al*, 1984). Behavioural (e.g., the difficulty of using a condom consistently in stable relationships) or biological (e.g., extension of HPV infection beyond the anatomical area covered by a condom) factors may account for this finding.

Our study has certain weaknesses but also some strengths. Unfortunately, we were able to search for relatively few mucosal HPV types, and no skin type. Viral load could not be assessed, but, on the basis of rather low hybridisation signals, we had the impression that viral copy numbers in men are generally lower than in women.

As three of seven studies were hospital-based, the determined prevalence of HPV DNA is not necessarily representative of that in the general male population. Differences in study design (i.e., population- *vs* hospital-based studies, the choice of various hospital wards, etc.) also obliged us to interpret between-country comparisons cautiously. The prevalence of penile HPV infection in our study is, however, positively correlated with cervical cancer incidence rates in the populations under study (i.e., highest in Brazil and Colombia and lower elsewhere) (Parkin *et al*, 1997).

Within-country comparisons of HPV-positive and negative men should not suffer from selection or recall bias, since all interviews were conducted prior to HPV-testing. Also reassuring is the close similarity, with respect to various indicators of sexual behaviour, between the 1143 husbands for whom HPV findings were available and the 778 ones who either refused to participate or had inadequate penile cell specimens. In order to increase the study power, husbands of control women and CC case women were pooled together in some analyses, on account of the similarity in most findings. Wives' case/control status was, however, always adjusted for and the consistency of findings was confirmed by stratified analyses. For instance, the OR for penile HPV infection was 1.5 for 51 *vs* 1 lifetime sexual partners among the husbands of control women only, and 1.4 for 3 *vs* 1 regular partners.

The present study is notable for the large number of husbands and their wives from a wide range of age groups and countries with different background risks of cervical cancer. Detailed information on sexual habits and accurate procedures for the collection and HPV-testing of penile cell specimens represent additional strengths. The relatively low within-couple agreement in HPV-positivity at a single point in time and the steady prevalence of penile HPV infection with age suggest that the natural history of HPV may differ between men and women. Despite their difficulty, new cross-sectional and prospective studies in men are warranted to provide a basis for designing effective HPV prevention strategies.
